# Parental responsiveness, demandingness, and emotional eating among Chinese college students: the mediating role of interpersonal trust

**DOI:** 10.3389/fpsyg.2026.1745219

**Published:** 2026-08-10

**Authors:** Huixian Zhang

**Affiliations:** School of Education, Wuhan College of Arts and Sciences, Wuhan, China

**Keywords:** Chinese college students, emotional eating, interpersonal trust, parental demandingness, parental responsiveness

## Abstract

**Objectives:**

This cross-sectional, online self-report study examined the associations between parenting styles and emotional eating among Chinese college students, with a specific focus on the mediating role of interpersonal trust. Emotional eating—a maladaptive strategy for coping with negative emotions such as anxiety, stress, and sadness—has been linked to psychological distress and adverse physical health outcomes. Guided by a health psychology perspective, we investigated how two core parenting dimensions, responsiveness and demandingness, relate to emotional eating, both directly and indirectly through interpersonal trust. A key methodological feature is that participants retrospectively recalled parenting experiences before age 16; this should be considered when interpreting the findings.

**Methods:**

Data were collected from 464 Chinese college students (*M* = 20.3 years; 82.5% female) via online self-administered questionnaires. Participants completed validated measures: the Parental Bonding Instrument (PBI; retrospective assessment of parenting before age 16), the Emotional Eating Scale, and the Interpersonal Trust Scale. Structural equation modeling (SEM) with bias-corrected bootstrap mediation (5,000 resamples) was used to examine direct and indirect effects of parenting styles on emotional eating, with interpersonal trust as the proposed mediator.

**Results:**

Paternal and maternal parenting styles showed high cross-parent consistency. Parental demandingness (overcontrol) was positively associated with emotional eating both directly (*β* = 0.303) and indirectly through lower interpersonal trust [indirect *β* = 0.085, 95% CI (0.027, 0.167)]. Parental responsiveness (care and autonomy support) was positively correlated with interpersonal trust but showed no significant direct or indirect association with emotional eating.

**Conclusion:**

Parental demandingness is associated with higher emotional eating, both directly and indirectly through reduced interpersonal trust. Parental responsiveness showed no significant direct association with emotional eating. These findings suggest that interventions targeting overcontrolling parenting and promoting interpersonal trust among college students could help mitigate emotional eating. Causal interpretations await longitudinal or experimental evidence.

## 1 Introduction

Emotional eating is defined as the behavior of regulating or alleviating emotional states through eating during emotional fluctuations (van Strien et al., 2007). Extensive research indicates that negative emotions such as anxiety, depression, and stress are closely associated with unhealthy eating behaviors (Liu et al., 2016; Cheung and Yip, 2016; Goossens et al., 2008), highlighting the crucial role of emotional regulation mechanisms in dietary control. College students face multiple pressures from academic stress, interpersonal relationships, and self-development. They experience frequent emotional fluctuations and possess limited coping abilities, making them more prone to emotional coping strategies like emotional eating. Therefore, college students are considered a crucial research population for exploring the psychological mechanisms of emotional eating.

## 2 Literature review and hypothesis development

### 2.1 Parenting as a multidimensional construct

Parenting is a complex construct that can be examined through two fundamental dimensions: responsiveness and demandingness (Lamborn et al., 1991; Alcaide et al., 2025b; Leung et al., 1998). Responsiveness refers to parental involvement, warmth, and support aimed at reinforcing the developing child’s individuality (Baumrind, 1991; Kassis et al., 2025). In contrast, demandingness encompasses parental strictness, supervision, and control to ensure that the child conforms to societal and family expectations (Steinberg et al., 1994). The combination of these two dimensions yields four prototypical parenting styles: authoritative (high responsiveness and high demandingness), authoritarian (high demandingness and low responsiveness), indulgent (high responsiveness and low demandingness), and neglectful (low responsiveness and low demandingness) (Baumrind, 1991; Lamborn et al., 1991).

Parenting styles can be analyzed either by examining the specific effects of each dimension (Alcaide et al., 2025b) or by investigating their combined configurations (Lamborn et al., 1991; Leung et al., 1998). Specific parenting practices, such as autonomy granting, reflect high parental responsiveness by encouraging adolescents to express individuality without coercion (Steinberg et al., 1994; Leung et al., 1998). Autonomy granting has been identified as particularly beneficial for helping children develop their individuality while adhering to societal values (Teuber et al., 2022).

### 2.2 Parental consistency in parenting styles

According to social exchange theory, individuals typically select partners who share similarities in educational attainment, values, and social class (Blau, 2016; Knox and Schacht, 2012). Such similarity not only reduces marital conflict and enhances communication quality but also lays a crucial foundation for co-parenting behaviors (Jacobson, 1981; Acitelli et al., 2001). Specifically, convergence between spouses in cultural tastes, behavioral preferences, and developmental goals fosters consistent educational expectations and parenting philosophies, promoting the formation of a “co-parenting alliance” (Li, 2022). Within this alliance framework, parents can collaboratively build parent–child relationships and create a family environment conducive to children’s healthy development (Edwards and Roff, 2016).

From a family systems theory perspective, the family is viewed as a complex and dynamic whole, where its subsystems and constituent elements exhibit close interdependence and mutual influence (Bowen, 1978). Due to shared living environments, cultural backgrounds, and long-term interaction histories, parenting behaviors within the same family often demonstrate significant non-independence (Kenny et al., 2006). Existing research confirms that parental marital satisfaction and dimensions of collaborative parenting are significantly positively correlated, further supporting the interconnectedness of elements within the family system (Liu et al., 2025). Parental similarity exerts a critical influence on the quality of their interactions and family functioning. Therefore, this study proposes:

*H1*: Fathers' parenting styles are significantly correlated with mothers' corresponding parenting styles.

### 2.3 Cultural variations in optimal parenting

Research primarily involving European-American families in the United States has identified the benefits of high demandingness combined with high responsiveness (i.e., authoritative parenting), whereas high demandingness combined with low responsiveness (authoritarian parenting) is associated with adolescent maladjustment (Darling and Steinberg, 1993; Lamborn et al., 1991; Steinberg et al., 1994). However, the optimal parenting style may vary across cultural contexts (Garcia et al., 2019; Pinquart and Gochaliyev, 2025). Studies with ethnic minorities in the US and families from Arab societies have identified benefits associated with authoritarian parenting, characterized by high demandingness and low responsiveness (Baumrind, 1972; Chao, 2001; Dwairy et al., 2006). A possible explanation is that, regardless of parenting style, children may still feel loved and appreciated within their families, and this emotional tie can lead to adherence to societal rules and positive developmental outcomes (Patil et al., 2025; Villarejo et al., 2024).

Research with Chinese families has traditionally suggested benefits associated with high demandingness, even when combined with low responsiveness (i.e., authoritarian parenting), particularly for academic success (Chao, 1994, 2001). These benefits have been attributed to cultural characteristics of Chinese collectivism and hierarchical relationships, where children are expected to obey their parents (filial piety), while parents are seen as responsible for governing, teaching, and disciplining their children through practices known as chiao shun and guan (Chen et al., 2024). Nevertheless, recent findings from families in China indicate the benefits of parental responsiveness, regardless of parental demandingness; parenting based on demandingness without responsiveness (authoritarian style) has been identified as negative for child development (Alcaide et al., 2025a; Chen et al., 2025). Similarly, research from Europe has highlighted the advantages of parenting styles based on responsiveness—such as authoritative and indulgent parenting—compared to styles characterized by low responsiveness, such as authoritarian and neglectful parenting (Garcia et al., 2019; Martinez-Escudero et al., 2023). Interestingly, among European families, parental responsiveness is related to benefits, whereas parental demandingness appears unnecessary (no differences were found between adolescents with authoritative and indulgent parents) and may even be detrimental in some cases (Villarejo et al., 2024).

### 2.4 Parenting and emotional eating

From a family systems theory perspective, the family constitutes the most central micro-ecosystem in an individual’s developmental journey, with parental parenting styles forming a crucial component of this system (Bowen, 1978). Extending this to emerging adults, retrospective perceptions of responsive parenting are expected to be associated with better emotion regulation and lower emotional eating in college students as well. The stable behavioral patterns exhibited by parents during childrearing shape the emotional atmosphere and interaction quality within the family environment, exerting a lasting influence on an individual’s socialization process and psychological adaptation (Liu et al., 2022).

Existing research indicates that parental responsiveness and demandingness exert profound effects on adolescents’ emotional regulation abilities, interpersonal trust levels, and dietary behaviors. Warm, caring, and supportive parenting (high responsiveness) promotes secure attachment and positive emotional regulation strategies in children, thereby reducing the risk of emotional eating. Conversely, controlling or neglectful parenting (high demandingness without responsiveness) may weaken an individual’s emotional regulation and self-control abilities, increasing the likelihood of emotional eating (Jiang and Zhou, 2020). Families of eating disorder patients often exhibit poor father-daughter communication, emotional detachment, and authoritarian fathering, alongside mothers who are overprotective yet lack warmth and acceptance (Cao et al., 2012). Therefore, this study proposes:

*H2*: Parental responsiveness negatively predicts emotional eating among college students, whereas parental demandingness positively predicts emotional eating.

### 2.5 Parenting, interpersonal trust, and emotional eating

Parental interaction in parenting styles significantly influences children’s development of interpersonal trust (Chen and An, 2019). Attachment theory posits that positive parent–child interactions foster secure attachment and promote children’s interpersonal trust (You et al., 2025). Conversely, disparagement and conflict in parenting may induce hostile attitudes in children, hindering trust formation (Zhao et al., 2022). Individuals chronically exposed to parenting characterized by high demandingness and low responsiveness, due to unmet emotional needs, are prone to develop insecure attachment patterns, leading to distrust of their surroundings. When facing stress, they tend to adopt negative emotion regulation strategies, making them prone to emotional dysregulation and problem behaviors. Individuals may cope with negative emotions through abnormal eating behaviors, where eating becomes a strategy to temporarily alleviate anxiety, depression, and other negative emotions (Brennan et al., 1998; O’Shaughnessy and Dallos, 2009; Faber et al., 2018).

Related research indicates that insecure attachment is present in 70 to 100% of individuals with eating disorders (O’Shaughnessy and Dallos, 2009); among healthy populations, insecure attachment is also closely associated with subclinical eating disorder symptoms (Bamford and Halliwell, 2009). Recent meta-analysis findings reveal a significant negative correlation between higher interpersonal trust derived from secure attachment and unhealthy eating behaviors (Faber et al., 2018). Therefore, this study proposes:

*H3*: Interpersonal trust mediates the relationship between parenting styles and emotional eating behaviors.

### 2.6 The present study and hypotheses

Based on the theoretical framework and empirical evidence reviewed above, the present study aims to examine the associations between parenting styles and emotional eating among Chinese college students, with a specific focus on the mediating role of interpersonal trust. Drawing on the two-dimensional model of parenting (responsiveness and demandingness) and integrating insights from family systems theory, social exchange theory, and attachment theory, this study seeks to achieve three primary objectives: (1) to investigate the consistency between paternal and maternal parenting styles within Chinese families; (2) to examine the direct effects of parental responsiveness and demandingness on emotional eating; and (3) to test whether interpersonal trust serves as a mediating mechanism linking parenting styles to emotional eating.

In line with the theoretical rationale and empirical evidence presented in the preceding sections, the following hypotheses were formulated:

*H1*: Fathers' parenting styles are significantly and positively correlated with mothers' corresponding parenting styles (i.e., care, encouragement of autonomy, and control).

*H2*: Parental responsiveness negatively predicts emotional eating among college students, whereas parental demandingness positively predicts emotional eating.

*H3*: Interpersonal trust mediates the relationship between parenting styles and emotional eating among college students. Specifically, parental demandingness is expected to undermine interpersonal trust, which in turn increases emotional eating tendencies, whereas parental responsiveness is expected to foster interpersonal trust, thereby reducing emotional eating.

Although the theoretical frameworks cited above were originally developed to explain child and adolescent outcomes, they extend to emerging adults’ retrospective reports for three reasons. First, parenting experiences shape internalized working models and emotion regulation strategies that persist into young adulthood. Second, retrospective perceptions—even if imperfect—reflect the individual’s subjective reality and directly influence current coping behaviors. Third, emotional eating often originates early in life and remains relevant during college years, when stress levels rise. Consistent with this logic, prior Chinese studies have successfully used the PBI to predict emerging adult outcomes (e.g., depression, anxiety, eating disturbances).

## 3 Methods and instruments

### 3.1 Participants

This cross-sectional study collected data from Chinese college students via an anonymous online questionnaire distributed through university networks. Participation was voluntary, and respondents provided informed consent before completion.

Data were collected in two waves: 426 questionnaires in the first wave (June 2–15, 2023) and 38 in the second wave (September 10, 2025) to increase sample size. All 464 returned questionnaires met quality criteria (≤10% missing, consistent reverse-coded responses, completion time ≥4 min based on a pilot test), so none were excluded. No statistical comparisons were performed between waves due to the small second wave (8.2%); the 27-month gap is a limitation (see Discussion).

Participants’ mean age was 20.3 years (SD = 1.6); 82.5% were female. Only-child status: 26.7%. Parents were married for 85.6% of participants. Father’s education: junior high (41.8%), high school (23.5%), primary (21.1%), college+ (13.6%). Mother’s education: junior high (38.8%), primary (31.5%), high school (18.5%), college+ (11.2%).

### 3.2 Measures

The questionnaire consisted of four sections assessing parental bonding, emotional eating, and interpersonal trust, along with demographic information (including gender, age, and socioeconomic status).

Parental bonding was assessed using the Chinese version of the Parental Bonding Instrument (PBI; Yang et al., 2009), which retrospectively measures parenting experiences before age 16. The instrument includes separate forms for mothers and fathers (each 23 items; 5-point Likert scale) and comprises three dimensions: care (11 items; warmth, understanding), encouragement of autonomy (6 items; respect for independence), and control (6 items; intrusive, overprotective behaviors). Sample items include “Spoke to me in a warm and friendly voice” (care), “Let me decide things for myself” (autonomy), and “Tried to control everything I did” (control). In this study, Cronbach’s *α* for maternal/paternal versions were: care 0.87/0.86, autonomy 0.88/0.87, control 0.79/0.80.

Following the responsiveness - demandingness framework (Baumrind, 1991), we mapped care and autonomy onto parental responsiveness (emotional warmth + autonomy support) and the control dimension onto parental demandingness. Notably, the PBI control subscale measures psychological control and overprotection, a specific facet of demandingness rather than broader behavioral monitoring (Barber, 1996). This mapping is supported by Chinese validation studies (e.g., Yang et al., 2009; two-factor PBI structure predicts outcomes consistent with Baumrind’s model). The PBI was preferred over alternatives (e.g., PAQ) because it is retrospective, validated in Chinese populations, and assesses mothers and fathers separately.

#### 3.2.1 Emotional eating scale

Emotional eating was assessed using the Emotional Eating Scale (EES) developed by Zhu et al. (2016). This 23-item scale measures individuals’ tendency to eat in response to various emotional states. The scale encompasses four dimensions: depression-related eating, anxiety-related eating, hostility-related eating, and positive emotion-related eating. Participants rated each item on a 5-point Likert scale ranging from 1 (never) to 5 (always), with higher total scores indicating a greater propensity for emotional eating. Representative items include: “When you feel depressed or discouraged, do you crave eating?”; “When you feel lonely, do you crave eating?”; and “When you feel tense, worried, or anxious, do you crave eating?” The scale demonstrated excellent reliability in this study, with a Cronbach’s *α* coefficient of 0.947.

#### 3.2.2 Interpersonal trust scale

Interpersonal trust was measured using the Chinese version of the Interpersonal Trust Scale (ITS) revised by Ding and Peng (2020), adapted from Rotter’s original scale (Rotter, 1967). This 10-item instrument assesses individuals’ general expectations about the trustworthiness of others’ verbal statements and promises. Items are rated on a 5-point Likert scale ranging from 1 (strongly disagree) to 5 (strongly agree), with higher total scores reflecting greater levels of interpersonal trust. Sample items include: “I often suspect that when people help me, there might be some hidden reason behind it” (reverse-scored); “Most people are honest primarily because they are afraid of being caught”; and “I think most people would lie in order to succeed.” In the present study, the scale showed acceptable internal consistency, with a Cronbach’s α coefficient of 0.69.

### 3.3 Data analysis

Data were analyzed using SPSS 27.0 and Mplus 8.3. Confirmatory factor analysis (CFA) was conducted to verify the structural validity of each questionnaire. Harman’s single-factor test was employed to examine common method bias. Correlations among variables were assessed using product–moment correlation analysis. The hypothesized mediation model was tested using the bias-corrected nonparametric percentile bootstrap method with 5,000 resamples (Hayes, 2013). Model fit was evaluated using standard fit indices, including *χ*^2^/df, CFI, TLI, RMSEA, and SRMR.

## 4 Results

### 4.1 Confirmatory factor analysis

To examine the structural validity of the core measures, confirmatory factor analysis (CFA) was conducted using Mplus 8.3. The model included four latent variables: parental responsiveness, parental demandingness, interpersonal trust, and emotional eating. Results indicated that the measurement model fit the data adequately: *χ*^2^/df = 3.24, CFI = 0.847, TLI = 0.837, RMSEA = 0.069, SRMR = 0.057. Standardized factor loadings for all items ranged from 0.485 to 0.908, and all were statistically significant (*p* < 0.001). Composite reliabilities (CR) of the latent variables ranged from 0.73 to 0.96, and average variance extracted (AVE) ranged from 0.50 to 0.70, all exceeding the recommended thresholds (CR > 0.70, AVE > 0.50), indicating good convergent validity for each scale.

### 4.2 Common method bias test

As all variables were measured using self-report questionnaires, the potential influence of common method bias (CMB) on the observed associations among variables was examined. Prior to data analysis, Harman’s single-factor test was conducted using exploratory factor analysis on all items. The results revealed 18 factors with eigenvalues exceeding 1, with the first factor accounting for 15.97% of the total variance—well below the recommended threshold of 40%. This indicates that common method bias did not substantially affect the findings of this study.

### 4.3 Descriptive statistics and correlation analysis

Table 1 presents the means, standard deviations, and bivariate correlations among the main study variables. Several notable patterns emerged from the correlation analysis.

**Table 1:** Descriptive statistics and correlations among key variables.

Variable	*M* ± SD	1	2	3	4	5	6	7	8
1. Father’s care	2.51 ± 0.42	1							
2. Maternal care	2.93 ± 0.59	0.63^**^	1						
3. Father encourages autonomy	2.95 ± 0.67	0.48^**^	0.43^**^	1					
4. Mother encourages autonomy	2.87 ± 0.67	0.41^**^	0.57^**^	0.65^**^	1				
5. Father’s control	1.82 ± 0.61	−0.44^**^	−0.35^**^	−0.27^**^	−0.23^**^	1			
6. Maternal control	1.94 ± 0.62	−0.41^**^	−0.44^**^	−0.18^**^	−0.32^**^	0.66^**^	1		
7. Interpersonal trust	3.40 ± 0.80	0.21^**^	0.22^**^	0.05	0.11^*^	−0.19^**^	−0.20^**^	1	
8. Emotional eating	2.37 ± 0.95	−0.11^*^	−0.09	−0.05	−0.02	0.23^**^	0.17^**^	−0.32^**^	1

*N* = 464; *p < 0.05, **p < 0.01. Same applies for Table 3. Control refers to the PBI control subscale, which measures parental overprotection and psychological control. In subsequent analyses, parental responsiveness is operationalized as the average of care and encouragement of autonomy; parental demandingness is represented by the control subscale alone.

First, significant consistency was observed between paternal and maternal parenting styles across corresponding dimensions. Strong positive correlations were found between fathers’ and mothers’ care (*r* = 0.63, *p* < 0.01), encouragement of autonomy (*r* = 0.65, *p* < 0.01), and control (*r* = 0.66, *p* < 0.01), providing preliminary support for H1.

Second, parental control (demandingness) was negatively associated with parental care and encouragement of autonomy (responsiveness). For instance, maternal control showed significant negative correlations with maternal care (*r* = −0.44, *p* < 0.01) and maternal encouragement of autonomy (*r* = −0.32, *p* < 0.01), suggesting that these two parenting dimensions are conceptually distinct.

Third, in terms of mean scores, participants reported higher levels of parental care (*M* = 2.51–2.93) and encouragement of autonomy (*M* = 2.87–2.95) compared to parental control (*M* = 1.82–1.94), indicating that the sample perceived their parents as predominantly responsive rather than demanding.

### 4.4 Relationships between parenting dimensions, interpersonal trust, and emotional eating

Following the operationalization described in the note to Table 2, composite scores were computed for parental responsiveness (aggregating care and encouragement of autonomy) and parental demandingness (using the control subscale alone). Table 3 presents the correlations among these composite variables.

**Table 2:** Demographic characteristics of the participants (*N* = 464).

Variable	Category	*n*	%
Gender	Male	81	17.5
	Female	383	82.5
Only child	Yes	124	26.7
	No	340	73.3
Parental marital status	Married	397	85.6
	Other (divorced / widowed / separated)	67	14.4
Father’s education	Primary school	98	21.1
	Junior high school	194	41.8
	High school	109	23.5
	College degree or above	63	13.6
Mother’s education	Primary school	146	31.5
	Junior high school	180	38.8
	High school	86	18.5
	College degree or above	52	11.2

**Table 3:** Correlations between parenting dimensions, interpersonal trust, and emotional eating.

Variable	*M* ± SD	1	2	3	4
1. Parental responsiveness	2.82 ± 0.47	1			
2. Parental demandingness	1.88 ± 0.56	−0.43^**^	1		
3. Interpersonal trust	3.40 ± 0.80	0.17^**^	−0.22^**^	1	
4. Emotional eating behavior	2.37 ± 0.95	−0.08	0.22^**^	−0.32^**^	1

Parental demandingness is operationalized using the PBI control subscale, which assesses overprotective and psychologically controlling parenting.

Parental responsiveness was positively correlated with interpersonal trust (*r* = 0.17, *p* < 0.01) but showed no significant association with emotional eating (*r* = −0.08, *p* > 0.05), providing partial support for the responsiveness component of H2. In contrast, parental demandingness was negatively correlated with interpersonal trust (*r* = −0.22, *p* < 0.01) and positively correlated with emotional eating (*r* = 0.22, *p* < 0.01), providing initial support for the demandingness component of H2.

### 4.5 Testing the mediating role of interpersonal trust

To test the hypothesized mediation model (H3), structural equation modeling (SEM) was conducted using Mplus 8.3 with 5,000 bootstrap resamples. Parental responsiveness and parental demandingness were entered as independent variables, interpersonal trust as the mediator, and emotional eating as the dependent variable. The model was saturated (i.e., all possible paths among the variables were estimated, resulting in zero degrees of freedom). By definition, a saturated model perfectly reproduces the sample covariance matrix; consequently, global fit indices (e.g., *χ*^2^, CFI, RMSEA, SRMR) necessarily take their perfect values (*χ*^2^ = 0, CFI = 1, RMSEA = 0) as a mathematical property, not as evidence of substantive model adequacy. Therefore, these fit indices are not substantively informative and should not be interpreted as indicating “perfect model fit.” Instead, the significance of indirect effects was evaluated using bias-corrected bootstrap confidence intervals, which provide valid inferences for mediation even in saturated models. Table 4 presents the direct, indirect, and total effects for both parenting dimensions. An indirect effect was considered statistically significant if its 95% bias-corrected bootstrap confidence interval did not contain zero.

**Table 4:** Mediation effects of interpersonal trust in the associations between parenting dimensions and emotional eating.

Path	Effect size	95% CI	*p*-value
Parental responsiveness			
Total effect	0.048	[−0.282, 0.355]	0.700
Direct effect	0.101	[−0.205, 0.406]	0.392
Indirect effect (via trust)	−0.053	[−0.146, 0.029]	0.226
Parental demandingness			
Total effect	0.392	[0.139, 0.630]	<0.001
Direct effect	0.303	[0.077, 0.537]	0.001
Indirect effect (via trust)	0.085	[0.027, 0.167]	0.018

*N = 464. Bootstrap resamples = 5,000. Bias-corrected confidence intervals are reported. An indirect effect is considered significant if the 95% CI does not contain zero. For parental demandingness, the indirect effect accounted for 21.7% of the total effect.

### 4.6 Sensitivity analysis

To examine whether the observed mediation effects were robust to potential confounding by demographic variables, we re-ran the structural equation model controlling for gender (dummy-coded: 1 = female, 0 = male), age (continuous), only-child status (1 = yes, 0 = no), parental marital status (1 = married, 0 = other), and father’s and mother’s education (ordinal). All covariates were entered as predictors of both interpersonal trust (mediator) and emotional eating (outcome), while the paths from parenting dimensions to the mediator and outcome were retained. The same bootstrap procedure (5,000 resamples) was applied.

As shown in Table 5, after controlling for demographic covariates, the pattern of mediation remained largely unchanged. Specifically, the indirect effect of parental demandingness on emotional eating via interpersonal trust remained significant [*β* = 0.078, 95% bias-corrected CI (0.021, 0.149), *p* = 0.026], accounting for 20.1% of the total effect. The direct effect of demandingness on emotional eating was also still significant [*β* = 0.289, 95% CI (0.065, 0.512), *p* = 0.003]. Parental responsiveness continued to show no significant indirect effect [*β* = −0.049, 95% CI (−0.138, 0.031), *p* = 0.241] or direct effect [*β* = 0.095, 95% CI (−0.198, 0.389), *p* = 0.378]. Among the covariates, gender was significantly associated with emotional eating (*β* = 0.18, *p* = 0.012), with females reporting higher levels, but none of the covariates altered the substantive conclusions. These findings indicate that the observed mediation effects are robust to demographic confounding.

**Table 5:** Mediation effects of interpersonal trust after controlling for demographic covariates.

Path	Effect size	95% CI	*p*-value
Parental responsiveness			
Total effect	0.046	[−0.279, 0.350]	0.715
Direct effect	0.095	[−0.198, 0.389]	0.378
Indirect effect (via trust)	−0.049	[−0.138, 0.031]	0.241
Parental demandingness			
Total effect	0.367	[0.122, 0.603]	<0.001
Direct effect	0.289	[0.065, 0.512]	0.003
Indirect effect (via trust)	0.078	[0.021, 0.149]	0.026

*N* = 464. Bootstrap resamples = 5,000. Covariates included gender, age, only-child status, parental marital status, and parents’ education. Bias-corrected confidence intervals are reported.

### 4.7 Summary of results

Three key findings emerged. First, supporting H1, paternal and maternal parenting styles were significantly and positively correlated across all three dimensions. Second, consistent with H2 (specifically the demandingness component), parental demandingness was positively associated with emotional eating both directly (*β* = 0.303) and indirectly via interpersonal trust [indirect *β* = 0.085, 95% CI (0.027, 0.167)], with the indirect effect accounting for 21.7% of the total effect. Third, contrary to the responsiveness component of H2, parental responsiveness showed no significant direct or indirect association with emotional eating. Together, these findings highlight the distinct roles of demandingness and responsiveness, with interpersonal trust serving as a specific mediating mechanism for the demandingness–emotional eating pathway.

## 5 Discussion

Using the responsiveness -demandingness framework, this study examined how parenting styles relate to emotional eating among Chinese college students via interpersonal trust. In discussion, our participants are referred to as “college students” or “emerging adults”; original terminology from cited studies is retained.

### 5.1 Consistency between paternal and maternal parenting styles

The results indicated that among college students, mothers and fathers showed a high level of consistency across corresponding parenting dimensions, supporting Hypothesis H1. Significant positive correlations were observed between paternal and maternal care (*r* = 0.63, *p* < 0.01), encouragement of autonomy (*r* = 0.65, *p* < 0.01), and control (*r* = 0.66, *p* < 0.01). This finding corroborates the cognitive consistency theory perspective that individuals tend to seek partners with similar attitudes in mate selection (Xu and Ye, 1999), aligning with inferences from marital matching theory and social exchange theory (Blau, 2016; Knox and Schacht, 2012).

Such similarity between parents facilitates the formation of a stable “co-parenting alliance,” enhancing parenting coordination and providing children with a consistent socialization environment for psychological and behavioral development (Li, 2022). From a family systems theory perspective, high consistency between the couple subsystem and the parenting subsystem is crucial for maintaining stable family functioning (Bowen, 1978). When parents share similar values, expectations, and parenting approaches, children receive coherent messages about behavioral norms and emotional expression, which may contribute to more adaptive developmental outcomes.

It is also possible that parenting consistency is shaped by bidirectional influences (e.g., child temperament or behavior), although our cross-sectional data cannot test this direction. Future longitudinal research could examine how parent–child interactions co-evolve over time.

### 5.2 Effects of parenting styles on college students’ emotional eating: comparison with previous literature on optimal parenting

The findings suggested distinct patterns for the two parenting dimensions. Parental responsiveness showed no significant direct association with emotional eating, whereas parental demandingness exhibited a significant positive correlation with emotional eating (*β* = 0.22, *p* < 0.01). These results offer partial support for Hypothesis H2: the demandingness component (positive prediction) was supported, whereas the responsiveness component (negative prediction) was not.

When compared with existing literature, the findings from this study suggest the benefits of parental responsiveness (in terms of care and autonomy granting) in fostering interpersonal trust and protecting against emotional eating, although the direct effect on emotional eating was not significant. Conversely, the findings related to parental demandingness appeared to show consistently less favorable outcomes, including lower interpersonal trust and higher emotional eating.

Importantly, the findings from this study do not align with some previous research that has suggested benefits of parental demandingness without responsiveness (i.e., authoritarian parenting) within collectivist contexts, particularly among Chinese families (Chao, 1994, 2001). These earlier studies argued that in Chinese cultural contexts characterized by filial piety, hierarchical relationships, and collectivist values, authoritarian parenting might be interpreted as parental concern and involvement, leading to positive developmental outcomes, especially academic achievement.

In contrast, the present findings are consistent with more recent studies conducted with Chinese families, where parental responsiveness has been identified as beneficial, while authoritarian parenting has been related to negative developmental outcomes (Alcaide et al., 2025a; Chen et al., 2025). This pattern of findings suggests a potential cultural shift in contemporary Chinese society toward more egalitarian family relationships and increased individualism, although some collectivist values—such as the importance of academic success not only for the child but also for the family—may still persist to some extent (Alcaide et al., 2025b; Chen et al., 2024).

It is possible that Chinese children and adolescents today feel loved and appreciated when parents express warmth and involvement, internalizing social norms without requiring authority based on coldness, strictness, and firmness. Instead, they may respond better to warmth, emotional support, and autonomy granting (Alcaide et al., 2025a; Patil et al., 2025). This interpretation aligns with broader societal changes in China, including urbanization, globalization, exposure to diverse cultural values, and shifts in family structures.

Interestingly, the results of this study bear similarity to those found in families in Western contexts, including the United States and particularly Europe. Parenting that is based on responsiveness yields optimal outcomes across cultural contexts, although the role of demandingness may vary. In the United States, authoritative parenting—characterized by high responsiveness combined with high demandingness—has been consistently associated with positive developmental outcomes (Baumrind, 1991; Darling and Steinberg, 1993; Lamborn et al., 1991; Steinberg et al., 1994). In European contexts, research has shown that positive outcomes can occur even without demandingness, with indulgent parenting (high responsiveness, low demandingness) producing comparable or even superior outcomes to authoritative parenting in some cases (Alcaide et al., 2025b; Garcia et al., 2019; Leung et al., 1998; Kassis et al., 2025).

The null direct effect of responsiveness warrants consideration. First, responsiveness may influence emotional eating only indirectly via unmeasured mechanisms (e.g., self-esteem, self-control). Second, the retrospective reports may attenuate observed effects, or the influence of parenting may weaken as individuals transition to emerging adulthood. Third, cultural shifts in contemporary China may render warmth and autonomy support less directly relevant to eating behaviors than to broader psychological well-being. Future research should test these possibilities.

### 5.3 Mediating role of interpersonal trust

The bootstrap mediation analysis indicated that interpersonal trust significantly mediated the relationship between parental demandingness and emotional eating, partially supporting Hypothesis H3. The indirect effect of demandingness on emotional eating through interpersonal trust was significant [*β* = 0.085, 95% CI (0.027, 0.167)], accounting for approximately 22% of the total effect.

This finding suggests that parental overcontrol and overprotection are negatively associated with college students’ sense of security and interpersonal trust levels as recalled from childhood. When parents are excessively controlling, children may internalize the message that others are untrustworthy or that relationships are conditional, leading to generalized distrust. When such individuals experience negative emotions, the absence of a stable emotional support system may increase their tendency to adopt maladaptive coping strategies, such as emotional eating.

This finding aligns with attachment theory and social cognitive models, which posit that low interpersonal trust forms a psychological foundation for emotional eating (Brennan et al., 1998). Meta-analytic evidence also supports a negative correlation between secure attachment-derived trust and unhealthy eating (Faber et al., 2018).

Conversely, parental responsiveness did not produce a significant indirect effect through interpersonal trust, suggesting that responsive parenting may operate through alternative pathways (e.g., self-esteem, self-control). Future research could explore these mechanisms to deepen understanding of how different parenting dimensions influence eating behaviors.

### 5.4 Theoretical and practical implications

By incorporating interpersonal trust as a social-cognitive mediator, this study provides a novel framework for understanding how family socialization processes shape emotional eating patterns among young adults. Theoretically, it advances precision by using the responsiveness-demandingness framework rather than ambiguous “positive/negative” labels, integrates family systems and attachment perspectives, and contributes evidence for cultural shifts in Chinese parenting—where responsiveness is beneficial and demandingness without it appears detrimental. Practically, interventions targeting parental demandingness (overcontrol) and fostering interpersonal trust among college students may help mitigate emotional eating. Family education should help parents reduce overcontrol and balance structure with warmth, while campus programs can build trust and adaptive coping skills. Notably, the null findings for responsiveness suggest that interventions should not rely solely on promoting warm parenting; directly addressing demandingness and trust may be more effective.

### 5.5 Limitations and future directions

Although this study illuminates the psychological mechanisms by which parenting styles may influence college students’ emotional eating, several limitations should be acknowledged.

First, the cross-sectional design limits causal inferences. While the proposed mediation model is theoretically grounded and aligns with recent longitudinal findings in the parenting literature, the directional relationships among variables cannot be definitively established with cross-sectional data. Longitudinal and experimental approaches are warranted to examine the bidirectional effects proposed in our model and to establish temporal precedence among parenting styles, interpersonal trust, and emotional eating.

Second, the sample characteristics constrain generalizability. The predominance of female participants (82.5%) and the focus on college students from specific regions in China limit the extent to which findings can be generalized to other populations. Future research should test the model’s applicability across diverse age groups, gender-balanced samples, and varied cultural contexts to establish the robustness and cultural specificity of the observed relationships. Importantly, we conducted a sensitivity analysis controlling for demographic variables (gender, age, only-child status, parental marital status, and parents’ education), which confirmed that the mediation effects remained unchanged (see Section 4.6). Thus, the high proportion of female participants, while limiting generalizability, does not appear to have biased the core findings.

Third, beyond interpersonal trust, other psychological constructs may be integral to understanding emotional eating. Emotion regulation strategies, self-esteem, self-control, and body image concerns represent promising candidates for future investigation. Multivariate mediation models could elucidate how multiple mechanisms operate simultaneously or sequentially in linking parenting to eating behaviors.

Fourth, the reliance on self-report measures introduces the possibility of response biases, and specifically retrospective recall bias. Participants were asked to recall parenting experiences that occurred before age 16. Retrospective reports may be influenced by current mood, subsequent parent–child relationship quality, or psychological adjustment (e.g., depression or anxiety), potentially leading to systematic over- or under-reporting of certain parenting behaviors. Although the Parental Bonding Instrument is designed for retrospective recall and has been validated in Chinese populations, causal inferences regarding the temporal direction from parenting to emotional eating remain tentative. Future research could incorporate prospective longitudinal designs or parent-reported measures to triangulate findings.

Fifth, the measurement of interpersonal trust yielded acceptable but modest internal consistency (*α* = 0.69). This value falls slightly below the conventional threshold of 0.70, which may have introduced measurement error and potentially attenuated the estimated indirect effects. Given that interpersonal trust was a key mediator in our model, this limitation warrants caution when interpreting the magnitude of the mediation effects. Future research should employ alternative or expanded measures of interpersonal trust (e.g., the full Rotter scale or context-specific trust measures) to ensure more reliable assessment of this construct.

Sixth, temporal discontinuity in data collection. Data were collected in two waves separated by 27 months (June 2023 and September 2025). Most participants (91.8%, 426/464) were from the first wave; a small second wave (*n* = 38) was added to increase sample size. No statistical comparisons were performed due to the small second wave. Unmeasured temporal confounds (e.g., changes in academic stress or pandemic-related mental health) may have influenced responses. Future research should use a single recruitment window.

Finally, while this study identified a potential cultural shift in contemporary Chinese parenting, direct measures of cultural values (e.g., individualism, collectivism, filial piety) were not included. Future research should incorporate such measures to directly test the role of cultural factors in moderating the relationships between parenting styles and child outcomes.

### 5.6 Conclusion

In conclusion, by conceptualizing parenting through the two-dimensional framework of responsiveness and demandingness and integrating family systems and social cognitive perspectives, this study contributes to a more nuanced understanding of how parenting styles relate to emotional eating in emerging adulthood. The findings demonstrate that parental demandingness is positively associated with emotional eating both directly and indirectly through its negative association with interpersonal trust, whereas parental responsiveness shows no significant direct effects on emotional eating in this sample.

These results diverge from earlier perspectives that emphasized the benefits of parental strictness in Chinese cultural contexts, while aligning with more contemporary evidence highlighting the benefits of parental warmth and support. The patterns observed in this study bear similarity to findings from both Western and European contexts, suggesting that responsive parenting may confer universal benefits, even as its manifestations are shaped by cultural context.

Taken together, the present findings provide preliminary empirical evidence that may inform targeted prevention strategies addressing the socio-emotional antecedents of unhealthy eating patterns among college students, with particular attention to the evolving family dynamics in contemporary Chinese society.

## Data Availability

The original contributions presented in the study are included in the article/supplementary material, further inquiries can be directed to the corresponding author.
